# Pons metabolite alterations in narcolepsy type 1

**DOI:** 10.1007/s10072-025-08009-w

**Published:** 2025-02-14

**Authors:** Micaela Mitolo, Fabio Pizza, David Neil Manners, Lucia Guidi, Annalena Venneri, Luca Morandi, Caterina Tonon, Giuseppe Plazzi, Raffaele Lodi

**Affiliations:** 1 Department of Medicine and Surgery, University of Parma, Parma, Italy; 2 Functional and Molecular Neuroimaging Unit, IRCCS Istituto delle Scienze Neurologiche di Bologna, Bologna, Italy; 3 Department of Biomedical and Neuromotor Sciences, University of Bologna, Bologna, Italy; 4 IRCCS Istituto delle Scienze Neurologiche di Bologna, Bologna, Italy; 5 Department for Life Quality Sciences, University of Bologna, Bologna, Italy; 6 Department of Life Sciences, Brunel University London, Uxbridge, UK; 7 Department of Biomedical, Metabolic and Neural Sciences, University of Modena and Reggio Emilia, Modena, Italy

**Keywords:** Narcolepsy type 1, Proton MR spectroscopy, Pons, Metabolic alterations

## Abstract

**Introduction:**

Narcolepsy type 1 (NT1) is a rare central sleep disorder characterized by a selective loss of hypocretin/orexin (hcrt)-producing neurons in the postero-lateral hypothalamus that project to widespread areas of the brain and brainstem. The aim of this study was to explore in a group of NT1 patients the metabolic alterations in the pons and their associations with disease features.

**Methods:**

Twenty-one NT1 patients (16 M) and twenty age-matched healthy controls (10 M) underwent a brain ^1^H MRS on a 1.5 T GE Medical Systems scanner. Metabolite content of N-acetyl-aspartate (NAA), choline (Cho), and myo-inositol (mI) were estimated relative to creatine (Cr), using LCModel 6.3. Clinical data were also collected with validated questionnaires, polysomnography, the Multiple Sleep Latency Test (MSLT), Cerebrospinal fluid hypocretin-1 (CSF hcrt-1) concentration and genetic markers.

**Results:**

NT1 patients compared with healthy controls showed lower NAA/Cr ratio (*p* = 0.007) and NAA/mI ratio (*p* = 0.011) in the pons. The Epworth Sleepiness Scale score showed a significant negative correlation with NAA/Cr content (*p* = 0.023), MSLT sleep latency a negative correlation with the mI/Cr ratio (*p* = 0.008), and sleep onset REM periods a positive correlation with the mI/Cr ratio (*p* = 0.027). CSF hcrt-1 levels were positively correlated with the NAA/Cr ratio (*p* = 0.039) and negatively with the mI/Cr ratio (*p* = 0.045) and the Cho/Cr ratio (*p* = 0.026).

**Conclusion:**

The metabolic alterations found in the pons of NT1 patients using the MR Spectroscopy technique were associated with subjective and objective disease severity measures, highlighting the crucial role of this biomarker in the pathophysiology of the disease.

## Introduction

Narcolepsy type 1 (NT1) is linked to a selective loss of hypocretin/orexin (hcrt)-producing neurons in the postero-lateral hypothalamus that project to widespread areas of the brain and brainstem, evidence supported in vivo by the deficiency of the neurotransmitter hypocretin-1 in the cerebrospinal fluid (CSF hcrt-1) of affected patients [1].

Previous MRI observations in NT1patients with vascular/non-specific brainstem lesions suggested that white matter alterations detected as hyperintensities on T2 weighted sequences in the pons, which receives descending output from hypothalamic hcrt neurons, play a crucial role in the pathogenesis of narcolepsy [2].

Proton MR Spectroscopy (^1^H MRS) is a non-invasive metabolic technique able to quantify several metabolites even in the absence of signal intensity or morphological changes detectable by using structural MR sequences [3]. To date, inconsistent findings have been found in the pons by small cohort studies that have investigated metabolite concentrations in NT1 patients [4, 5], but these studies have not taken into account possible associations with clinical, polysomnographic, and CSF hcrt-1 levels.

The aim of this study was to fill this gap by clarifying, in a larger homogeneous cohort of NT1 patients, the metabolic alterations in the pons and their associations with disease features.

## Methods

### Participants

Twenty-one consecutive drug-naive, adult NT1patients (16 males and 5 females; mean age = 41.75 ± 16.46 years) were enrolled in this study.

Twenty age-matched healthy controls (10 males and 10 females; mean age 37.72 ± 17.65 years), were also recruited. Exclusion criteria were as follows: other sleep disorders; excessive daytime sleepiness (EDS); neurological diseases; psychiatric disorders; alcohol, or substance abuse; congenital or inherited diseases; chronic pulmonary/respiratory disease, or heart disease; MRI contraindications and structural brain lesions based on MR imaging. Demographic and clinical data of participants were reported in Table 1.

## Brain MRI protocol

**Fig. 1:**
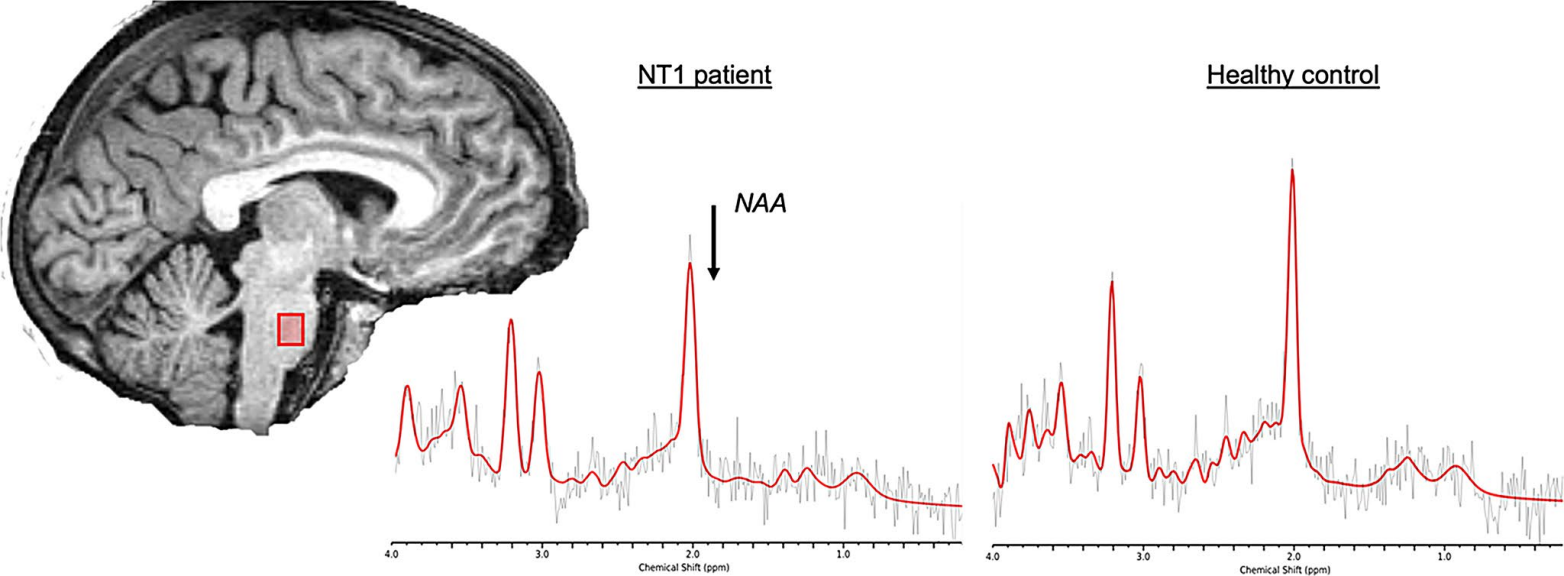
Localization of the pons MRS voxel (volume = 1.78 ± 1.11 ml; PRESS localization sequence, PROBE, TR = 1500 ms, TE = 40 ms, 512 FIDs) on the sagittal view of a partecipant’s T1-w image and two representative spectra, showing reduced NAA content in an NT1 patient (on the left) compared with ahealthy control (on the right). *NAA: N-acetyl-aspartate; ppm = parts per million*

The MRI protocol (1.5 T) included 3D T1-weighted FSPGR images (TR = 12.5 ms, TE = 5.1 ms, TI = 600 ms, 25.6 cm^2^ FOV; 1 mm^3^ isotropic voxels), T2 weighted sequences, FSE T2,and single voxel ^1^H spectra obtained from the pons (Volume Of Interest, VOI = 1.78 ± 1.11 ml, mean ± SD, Fig. 1) using the three planes of high resolution 3D T1-w sequence to optimize localization. Suppressed-water proton MR spectra were acquired using the PRESS localization sequence (PROBE) with the following parameters: TR = 1500 ms, TE = 40 ms, and averaging 512 FIDs for each acquisition [6]. Signal-intensity or major morphological brain alterations in patients and controls were excluded by an expert neuroradiologist (RL).

## Proton MR spectra analysis

Spectral analyses were performed using version 6.3 of LCModel. N-acetyl-aspartate (NAA), choline (Cho) and myo-Inositol (mI) content was evaluated and expressed relative to creatine (Cr). NAA content was also expressed relative to mI and Cho. The quality of each ^1^H MR spectrum was assessed according to standardized quality criteria [3, 7], both by visual inspection (CT) and by considering Signal to Noise Ratio, SNR, and Full Width at Half Maximum, FWHM (subjects were excluded if SNR < 3 and FWHM > 8). The quality of post-processing model fitting was evaluated through LCModel estimated fitting error, considering a SD > 20% as an exclusion criterion for metabolite evaluation.

### Statistical analysis

Normality of distributions was assessed with the Shapiro-Wilk test, T-test analyses were used for group comparisons and correlation analyses using Pearsons’s correlation were carried out between spectra data and clinical variables using the software Statistical Programme for Social Sciences (SPSS v.28).

## Results

NT1 patients and healthy controls were comparable for sex and age. No MR images nor proton MR spectra were excluded because of low quality, signal intensity or morphological changes either in the patient or in the healthy control groups.

There was a significantly lower NAA/Cr ratio (*p* = 0.007) in the pons of NT1 patients (mean ± sd, 2.34 ± 0.42) compared with healthy controls (2.77 ± 0.54), as well as of the NAA/mI ratio (*p* = 0.011, 1.62 ± 0.34 in NT1 patients, 2.12 ± 0.77 in healthy controls) (Table 1; Fig. 1).The ESS (Epworth Sleepiness Scale) score showed a significant negative correlation with NAA/Cr content (*r*=-0.493, *p* = 0.023). MSLT(Multiple Sleep Latency Test) sleep latency was negatively correlated with the mI/Cr ratio (*r*=-0.560, *p* = 0.008), and sleep onset REM periods were positively correlated with the mI/Cr ratio (*r* = 0.481, *p* = 0.027). CSF hcrt-1 levels were positively correlated with the NAA/Cr ratio (*r* = 0.519, *p* = 0.039), and negatively with the mI/Cr ratio (*r*=-0.508, *p* = 0.045) and the Cho/Cr ratio (*r*=-0.554, *p* = 0.026).

## Discussion

In this ^1^H-MRS study we found that NT1 patients present specific alterations of metabolites in the pons, and these changes are associated with disease severity measures and the levels of CSF hcrt-1.

Only two previous studies have explored pons alterations with the MRS technique in small samples of narcolepsy patients, with unclear results [4, 5]. Ellis and colleagues found no evidence of loss of neurons or of gross biochemical abnormalities in the ventral pons in a group of twelve idiopathic narcolepsy patients with cataplexy [4]. In contrast, Bican and colleagues showed significantly lower NAA/Cho ratios and higher Cho/Cr ratios in a small group of ten narcolepsy patients when compared with a healthy control group [5].

Cataplexy, the most specific clinical feature of NT1, has been shown to be associated with increased neural activity in supra-pontine centers that physiologically process emotions and rewards [8]; in addition, cataplexy-related increased activity has been reported in ponto-mesencephalic regions in the proximity of the locus coeruleus and midline pontine regions [8]. Areas within the pons are particularly important for the regulation of sleep, in particular the transition between non-REM and REM sleep and its concomitant atonia [9]. There is a complex system involving the pons and the hypothalamus that is thought to govern REM sleep on and off transitions [10].

Therefore, disruptions in the pons, which receive descending output from hypothalamic HCRT neurons, could play a crucial role in the pathogenesis of narcolepsy [2]. Moreover, the associations we found between metabolic pons alterations and subjective and objective sleepiness measures along with CSF hcrt-1 levels contribute valuable evidence in support of a crucial role of pons disruption in this disease.

NT1 diagnosis relies on polysomnographic data or the documentation of CSF hypocretin deficiency, the latter requiring a lumbar puncture. Previous studies have demonstrated that Proton MR Spectroscopy can be used to determine prognosis and to monitor treatment response in other central nervous system disorders [3]. Considering the significant association that we found in NT1 patients between metabolic pons alterations and other NT1 biomarkers, such as CSF hcrt-1 levels or objective sleepiness measures, future studies may evaluate the role of ^1^H-MRS in NT1 assessment. Indeed, the association we found between pons metabolites and CSF hcrt-1 levels suggests a future role for ^1^H-MRS as disease marker in the diagnosis of this condition and in the assessment of its severity, but also in monitoring response under orexin agonist treatments.

^1^H-MRS is a non-invasive, relatively fast, technique that, however, is not widely available in all neuroradiology departments (i.e. necessary hardware or knowledge is required for image acquisition). The technique provides useful molecular information related to specific brain areas of interest and can be usefully adopted in longitudinal studies of larger samples, in order to clarify in vivo the biological mechanisms underpinning narcolepsy, thereby shedding light on its role in prognosis and monitoring treatment response in this condition.

**Table 1:** Demographic, clinical and brain MRS data of the study samples

		NT1patients	Healthy controls	*p*-value
Demographics	**N**	21	20	
	**Gender (M/F)**	5 / 16	10 / 10	0.082
	**Age (mean ± sd)**,** y**	41.8 ± 16.5	37.7 ± 17.7	0.845
Clinical features	**EDS onset age**,** y**	22.05 ± 9.90	-	-
	**EDS duration**,** y**	19.52 ± 16.04	-	-
	**CATA onset age**,** y**	22.29 ± 9.60	-	-
	**CATA duration**,** y**	16.95 ± 14.25	-	-
	**CATA-CGI**	5.00 ± 1.61	-	-
	**Age at onset**,** y**	21.29 ± 9.98	-	-
	**Disease duration**,** y**	20.29 ± 16.81	-	-
	**Paralysis (yes/no)**	13/8	-	-
	**Hallucinations (yes/no)**	17/4	-	-
	**Disturbed sleep (yes/no)**	19/2	-	-
	**Clinical RBD (yes/no)**	10/ 11	-	-
	**ESS**	16.33 ± 3.90	-	-
	**MSLT-SL, min**	3.50 ± 2.43	-	-
	**MSLT-soremps**	4.19 ± 0.93	-	-
	**PSG-SE%**	81.30 ± 8.09	-	-
	**CSF HCRT** (pg/ml)	30.84 ± 44.52	-	-
	HLA-DQB1*0602 (yes/no)	19 / 2	-	-
Brain MRS	**NAA/Cr**	2.34 ± 0.42	2.77 ± 0.54	0.007*
	**mI/Cr**	1.48 ± 0.32	1.41 ± 0.38	0.521
	**NAA/mI**	1.62 ± 0.34	2.12 ± 0.77	0.011*
	**Cho/Cr**	0.62 ± 0.13	0.69 ± 0.14	0.109
	**NAA/Cho**	3.86 ± 0.81	3.99 ± 0.57	0.575

T1: narcolepsy type 1; EDS: excessive daytime sleepiness; CATA: cataplexy; CGI: Clinical Global Impression; RBD: Rapid eye movement, REM, sleep Behavior Disorder; ESS: Epworth Sleepiness Scale; MSLT-SL: multiple sleep latency test, sleep latency; MSLT-soremp:multiple sleep latency test, sleep onset REM periods; PSG:-SE%:polysomnography sleep efficiency; CSF: cerebrospinalfluid; HCRT: hypocretin/orexin; NAA: N-acetyl-aspartate; Cr: creatine; Cho: choline; mI: myo-Inositol

## Data Availability

All data relevant to this study have been disclosed in the manuscript. Further information may be shared upon request.
